# Traumatic brain injury in African urban centers: The role of road traffic accidents, outcomes, and health-system factors: a systematic review

**DOI:** 10.1186/s12883-026-05263-z

**Published:** 2026-08-08

**Authors:** Godswill Uzoechina, Abdulbasit Opeyemi Muili, Vidushi Joshi, Abbas F. Abdul Hussein, Edun Mariam Tolulope, Abdullahi Adekola, Grace Ogunlade, Opemipo Aiku

**Affiliations:** 1 Faculty of Clinical Sciences, University of Nigeria Teaching Hospital, Enugu, Nigeria; 2 Department of Medicine, Ladoke Akintola University of Technology, Ogbomosho, Nigeria; 3 China Medical University, Shenyang, China; 4 Department of Neurosurgery, College of Medicine, Babylon University, Al-Hillah, Iraq; 5 Department of Medicine and Surgery, University of Ilorin College of Health Sciences, Ilorin, Nigeria; 6 Department of Physiology, University of Ilorin, Ilorin, Nigeria; 7 Department of Medicine and Surgery, Afe Babalola University, Ekiti State, Ado-Ekiti, Nigeria

**Keywords:** Traumatic brain injury, Road traffic accidents, Neurosurgery, Africa, Urban health, Mortality

## Abstract

**Background:**

Traumatic brain injury (TBI) is a major cause of death and disability in low- and middle-income countries, particularly in rapidly urbanizing regions of Sub-Saharan Africa. Road traffic accidents (RTAs), especially motorcycle-related crashes, represent a leading mechanism of injury in many African urban settings. Limited access to neurosurgical care, neuroimaging, critical care services, and organized trauma systems may adversely affect patient outcomes. This systematic review synthesized evidence on outcomes and health-system factors among TBI cohorts in African urban centres where RTAs constitute a major mechanism of injury.

**Methods:**

PubMed and Google Scholar were systematically searched for studies published between January 2010 and May 2025. Eligible studies included observational studies, clinical audits, and hospital-based registries reporting outcomes among TBI patients treated in African urban centres. Primary outcomes were mortality and functional outcomes, including Glasgow Outcome Scale (GOS) or Glasgow Outcome Scale–Extended (GOSE) measures. Secondary outcomes included morbidity, length of hospital stay, and factors influencing access to neurosurgical care. Risk of bias was assessed using the Newcastle–Ottawa Scale tool. Owing to substantial clinical and methodological heterogeneity, findings were synthesized narratively without meta-analysis.

**Results:**

Thirteen studies from eight Sub-Saharan African countries were included. Study populations were predominantly composed of young adult males (mean or median age 28–34.5 years; 67–90% male), and RTAs were reported as the leading mechanism of injury in most settings. Severe TBI (Glasgow Coma Scale ≤ 8) was consistently associated with poor outcomes, with reported mortality ranging from approximately 25.8% to 47% among severe-injury cohorts. Predictors of adverse outcomes included low admission Glasgow Coma Scale score, hypotension, hypoxia, polytrauma, and delayed presentation to definitive care. Access to computed tomography (CT), intensive care unit (ICU) services, and neurosurgical intervention varied substantially across centres. Financial barriers frequently contributed to discharge against medical advice (DAMA), potentially compromising outcomes. Long-term functional and neuropsychological outcomes were infrequently reported. Most studies were judged to be at moderate risk of bias, with common limitations including retrospective study designs, single-centre recruitment, limited adjustment for confounding, and incomplete long-term follow-up.

**Conclusions:**

TBI remains a major cause of mortality and disability in African urban centres, with RTAs representing a dominant mechanism of injury in most included cohorts. Poor outcomes were associated with severe injury, delayed access to care, limited neuroimaging and critical care capacity, and financial barriers to treatment. The available evidence is constrained by heterogeneous outcome reporting and predominantly observational study designs. Future multicentre prospective studies with standardized outcome measures and longer-term follow-up are needed to better characterize outcomes and guide improvements in trauma and neurocritical care systems across Sub-Saharan Africa.

**Supplementary Information:**

The online version contains supplementary material available at https://doi.org/10.1186/s12883-026-05263-z.

## Introduction

Traumatic brain injury (TBI) is a major cause of death and disability worldwide and represents a substantial public health challenge across all age groups. It is caused by an external mechanical force to the head and may result from road traffic accidents (RTAs), falls, interpersonal violence, sports-related injuries, and other forms of trauma [1, 2]. Globally, an estimated 27–69 million individuals sustain a TBI annually, with more than 50 million new cases occurring each year [3, 4]. The burden of TBI extends beyond acute mortality, contributing significantly to long-term neurological disability, cognitive impairment, reduced quality of life, and considerable socioeconomic costs for patients, families, and healthcare systems [5, 6].

The epidemiology of TBI varies considerably across regions. In many high-income countries, falls have become the leading cause of TBI, particularly among older adults, reflecting demographic transitions and improvements in road safety measures [7, 8]. In contrast, road traffic accidents remain a dominant mechanism of TBI in many low- and middle-income countries, particularly in Sub-Saharan Africa, where rapid urbanization, increasing motorization, inadequate road infrastructure, weak enforcement of traffic regulations, and limited access to emergency medical services contribute to a disproportionately high burden of injury [9, 10].

Young adult males are consistently identified as the population most affected by TBI in African settings, largely because of their greater exposure to road traffic environments and occupational mobility [11, 12]. Motorcycle-related crashes represent an especially important contributor to neurotrauma in many urban centres, where commercial motorcycle transportation is widely used, and helmet compliance remains suboptimal [13]. Consequently, TBI imposes substantial clinical, social, and economic burdens in regions that often face significant resource constraints.

Outcomes following TBI are strongly influenced by both injury severity and healthcare system capacity. While established clinical predictors such as low Glasgow Coma Scale (GCS) score, hypotension, hypoxia, and polytrauma are associated with worse prognosis, access to timely neuroimaging, neurosurgical care, intensive care services, and organized trauma systems also play a critical role in determining patient outcomes [14–16]. In many African urban centres, limitations in prehospital care, delayed presentation, shortages of neurosurgical personnel, restricted access to computed tomography (CT), and substantial out-of-pocket healthcare costs may adversely affect survival and functional recovery [17, 18].

Although previous reviews have examined traumatic brain injury and road traffic injuries in Sub-Saharan Africa, no recent review has specifically synthesized outcomes and health-system factors among urban African TBI cohorts in which road traffic accidents constitute a major mechanism of injury. Given the continuing expansion of urban populations and the increasing burden of road traffic trauma across the region, a focused synthesis of available evidence is needed to better characterize outcomes and identify potentially modifiable health-system barriers.

Therefore, this systematic review aimed to synthesize the available evidence on mortality, functional outcomes, and health-system factors among patients with traumatic brain injury treated in African urban centres, with particular emphasis on settings where road traffic accidents represent a major mechanism of injury. We hypothesized that poor outcomes would be associated not only with greater injury severity but also with limitations in access to timely trauma, neurosurgical, and critical care services.

## Materials and methodology

### Protocol and registration

The protocol for this systematic review was prepared in line with PRISMA guidelines and is intended for registration in PROSPERO; however, final registration was pending at the time of manuscript submission due to ongoing author approval. The review adhered to the Cochrane Handbook for Systematic Reviews of Interventions and followed the PRISMA (Preferred Reporting Items for Systematic Reviews and Meta-Analyses) guidelines to ensure rigorous and standardized reporting.

### Eligibility criteria

Studies were eligible if they reported traumatic brain injury cohorts from African urban centres in which road traffic accidents constituted a major mechanism of injury or where mechanism-specific data were reported. Only primary studies (cohort, retrospective, prospective, cross-sectional, case–control) between 2010 and 2025 were eligible. The primary outcomes were mortality and functional outcomes (e.g., Glasgow Outcome Scale), and secondary outcomes included morbidity, duration of hospital stay, and time to neurosurgical intervention. Studies conducted outside African urban centres, studies not focused on traumatic brain injury, and studies without reportable clinical outcomes were excluded. For this review, “urban centers” were defined as studies conducted in tertiary or regional referral hospitals located in major metropolitan or city settings, with predominantly urban catchment populations.

### Search strategy

A comprehensive search was conducted in PubMed, Google Scholar, and African trauma registries (1 January 2010–18 May 2025). The following components were included in the search string: MeSH terms or other subject terms and synonyms and free-text keywords, tailored to each database. The search strategy was peer-reviewed by an information specialist according to PRESS guidelines. Key terms used included:

· “Traumatic Brain Injury” OR “TBI.”

· “Road Traffic Accidents” OR “Motor Vehicle Accidents.”

· “Neurosurgery” OR “Neurosurgical Trauma.”

· “Africa” OR names of specific countries

· “Urban centers” OR “city” OR “metropolitan.”

· “Outcomes” OR “mortality” OR “Glasgow Outcome Score.”

Only English-language studies were included.

After removal of duplicates, records were screened by title and abstract, followed by full-text review. A total of 13 studies met the inclusion criteria for final synthesis. In addition to PubMed and Google Scholar, African trauma registries, including the Malawi Trauma Registry and institutional hospital trauma databases where accessible, and reference lists of relevant studies were searched by hand for any other potentially includable papers. While Embase and Scopus are typically considered for systematic reviews, our search strategy was oriented towards sources that index the majority of African clinical literature. No further studies that met the inclusion criteria were identified through additional screening. Search strategy is documented in the Supplementary File 1.

### Study selection and data extraction

Titles and abstracts were independently screened by two reviewers. Full texts of potentially eligible studies were then retrieved and assessed independently by the same reviewers against the predefined eligibility criteria. Any disagreements at either the screening or full-text review stage were resolved through discussion and consensus; where necessary, a third reviewer was consulted.

Data were extracted using a standardized data extraction form. Extracted variables included study characteristics (author, year, and country), study design, sample size, patient demographic characteristics, clinical features, injury severity measures, follow-up duration, and reported outcomes, including mortality, functional outcomes, morbidity, and length of hospital stay.

### Risk of bias assessment

The Newcastle–Ottawa Scale was used to assess risk of bias because all included studies were observational cohort, registry, or cross-sectional designs reporting clinical outcomes of traumatic brain injury following road traffic accidents.

### Data synthesis

A narrative summary was conducted for study design, patient demographics, interventions, health-system factors, and outcomes. Although RTA-related TBI is the focus of the review, many of the included studies recruited mixed-mechanism TBI populations. These studies were retained if the mechanism of injury was predominantly road traffic accidents (usually > 50% of cases) and if outcomes were provided for the whole cohort without stratification by mechanism. This is also reflective of the configuration of available trauma datasets in many African urban centers, where mechanism-specific stratification is not commonly available. A quantitative meta-analysis was not conducted due to significant heterogeneity in study design, patient population, definition of outcomes, duration of follow-up, and reporting of mechanism-specific outcomes. Several reports contained mixed-mechanism TBI cohorts without stratified results for RTA-related cases, which also precluded the ability to pool quantitative estimates. Thus, the narrative synthesis method was considered methodologically more appropriate.

**Fig. 1:**
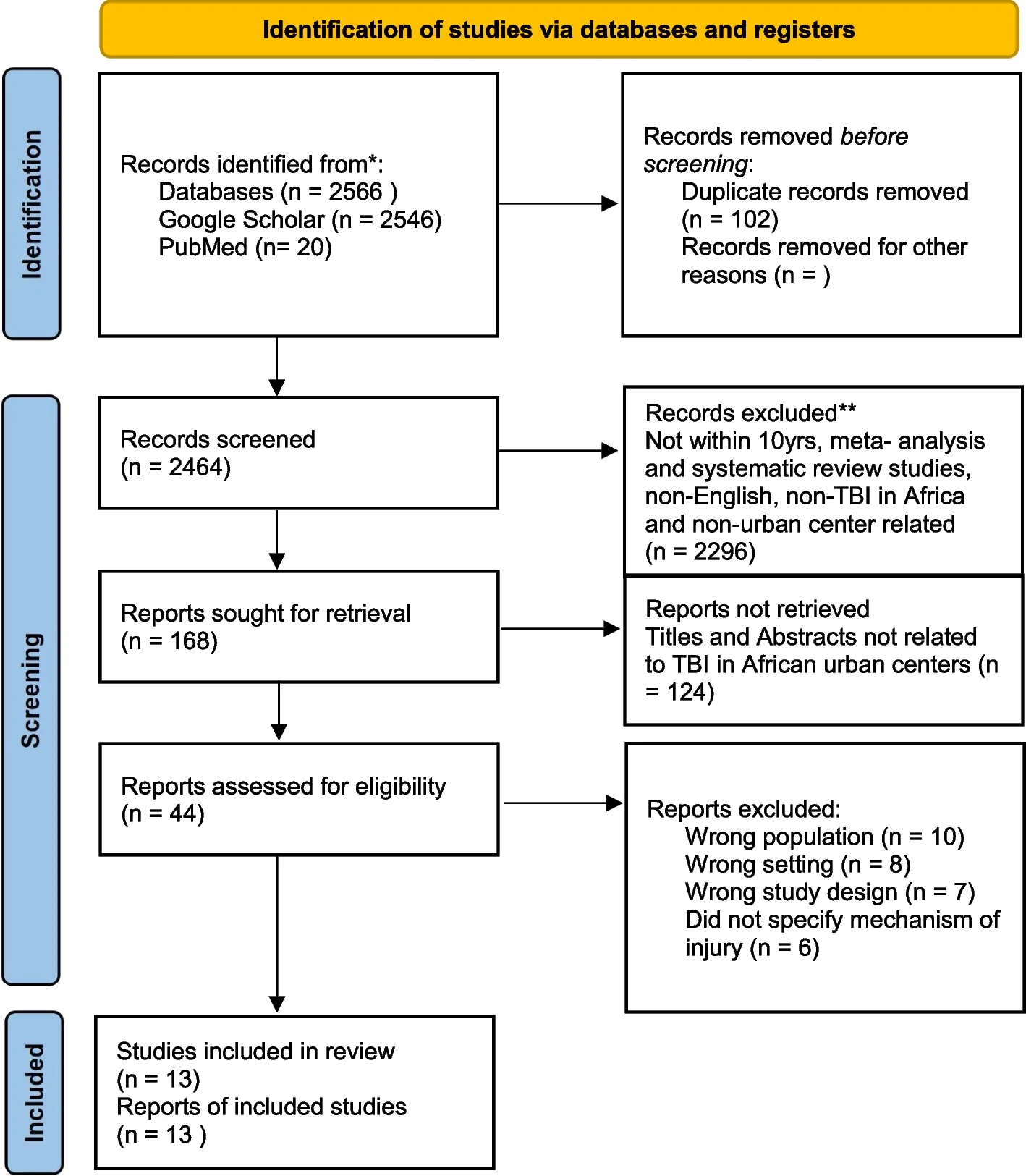
PRISMA flow diagram showing the process of study identification, screening, eligibility assessment, and final inclusion in the systematic review

The study selection process and reasons for exclusion are detailed in Fig. 1 (PRISMA flow diagram).

## Results

### Study selection

Records were identified from electronic databases, including Google Scholar (*n* = 2,546) and PubMed (*n* = 20), yielding a total of 2,566 records. After removal of duplicates (*n* = 102), 2,464 records remained for title and abstract screening.

Of these, 2,296 records were excluded as they did not meet the inclusion criteria, including studies published outside the 2010–2025 period, non-English language articles, studies not focused on traumatic brain injury, studies conducted outside African settings, non-urban hospital-based studies, and systematic reviews or meta-analyses.

A total of 168 full-text reports were sought for retrieval; however, 124 could not be obtained due to irrelevance to traumatic brain injury in African urban centres. Forty-four full-text articles were assessed for eligibility.

Of these, 31 studies were excluded for the following reasons: wrong population (*n* = 10), wrong setting (*n* = 8), wrong study design (*n* = 7), failure to specify mechanism of injury (*n* = 6). Ultimately, 13 studies met the inclusion criteria and were included in the final qualitative synthesis.

### Study inclusion and characteristics

This review included 13 studies from eight African countries, viz. Uganda (*n* = 3), Tanzania (*n* = 3), South Africa (*n* = 1), Cameroon (*n* = 1), Ethiopia (*n* = 1), Nigeria (*n *= 2), Botswana (*n* = 1), and Malawi (*n* = 1).

Study designs included four prospective cohorts [14, 16, 17, 19], one prospective registry analysis [12], and eight retrospective cohorts or observational studies [13, 15, 18, 20–24]. The sample sizes ranged from 100 to 2,149 participants across all studies; patients were predominantly young adults (median/mean age ≈ 28–34.5 years) and male (median proportion male ≈ 67–90%). The total sample size across included studies was 7,298 participants.

Road traffic accidents (RTAs) were the primary TBI mechanism (range 34.6–100% of cases), with motorcycle crashes (MCCs) cited as a major contributor in urban centers, ranging from 27.6–58.8% of RTA cases [15, 17, 18, 23]. Assault, falls, and other mechanisms were less frequent, although one large Nigerian study (Idowu 2014) reported assault as the leading cause (42%). A consistent pattern emerged: moderate and severe injuries (GCS ≤ 12 and GCS ≤ 8, respectively) constituted a significant proportion of admissions and were associated with markedly worse outcomes, though reporting of severity was heterogeneous.

Key characteristics, participant demographics, and risk of bias assessments are summarized in Table 1.

**Table 1:** Characteristics of included studies and risk of bias assessment

S/N	Author(s), Year	Country	Study Design	Sample Size (n)	Participant Demographics	RoB Tool	Overall RoB
12	Staton et al., 2017 [12]	Tanzania	Prospective registry/observational	893	Mean age 32.1; 80% male	Newcastle–Ottawa Scale	Moderate
13	Groshi & Enicker, 2022 [13]	South Africa	Retrospective cohort (severe TBI)	100	Mean age 29.5 ± 14.1; 85% male	Newcastle–Ottawa Scale	High
14	Buh et al., 2023 [14]	Cameroon	Prospective observational cohort	160	Median age 32 (IQR 26–39); 90% male	Newcastle–Ottawa Scale	Moderate
15	Boniface et al., 2017 [15]	Tanzania	Retrospective observational	627	Mean age 32.4; 86% male	Newcastle–Ottawa Scale	Moderate
16	Kalanzi et al., 2023 [16]	Uganda	Cross-sectional (severe RTC-TBI)	160	Median age 32 (IQR 25–39); 73.1% male	Adapted Newcastle–Ottawa Scale	Moderate
17	Chalya et al., 2012 [17]	Tanzania	Prospective hospital-based cohort	1,678	Mean age 29.45 ± 24.22; 67.2% male	Newcastle–Ottawa Scale	High
19	Eaton et al., 2017 [20]	Malawi	Retrospective cohort (registry)	280	Mean age 28.8 ± 16.3; 80.5% male	Newcastle–Ottawa Scale	Moderate
43	Mkubwa et al., 2022 [25]	Botswana	Retrospective ICU cohort	137	Mean age 34.5 ± 12.0; 83.2% male	Newcastle–Ottawa Scale	High
21	Bangirana et al., 2019 [19]	Uganda	Prospective observational cohort	171	Mean age 33.3 ± 13.4; 83% male	Newcastle–Ottawa Scale	Moderate
18	Kebede et al., 2024 [18]	Ethiopia	Multi-center retrospective cohort	402	Median ~ 30 yrs (IQR 21.5–43.5); 74.9% male	Newcastle–Ottawa Scale	High
22	Onyemaechi et al., 2020 [21]	Nigeria	Retrospective observational cohort	421	Mean age 34.4 ± 14.6; 76.7% male	Newcastle–Ottawa Scale	Moderate
23	Idowu & Akinbo, 2014 [22]	Nigeria	Retrospective observational (registry)	2,149	Mean age 31; M:F 3.1:1	Newcastle–Ottawa Scale	Moderate
24	Tran et al., 2015 [23]	Uganda	Retrospective chart review (severe TBI)	120	Predominantly male, modal 15–29 yrs	Newcastle–Ottawa Scale	Moderate

**Fig. 2:**
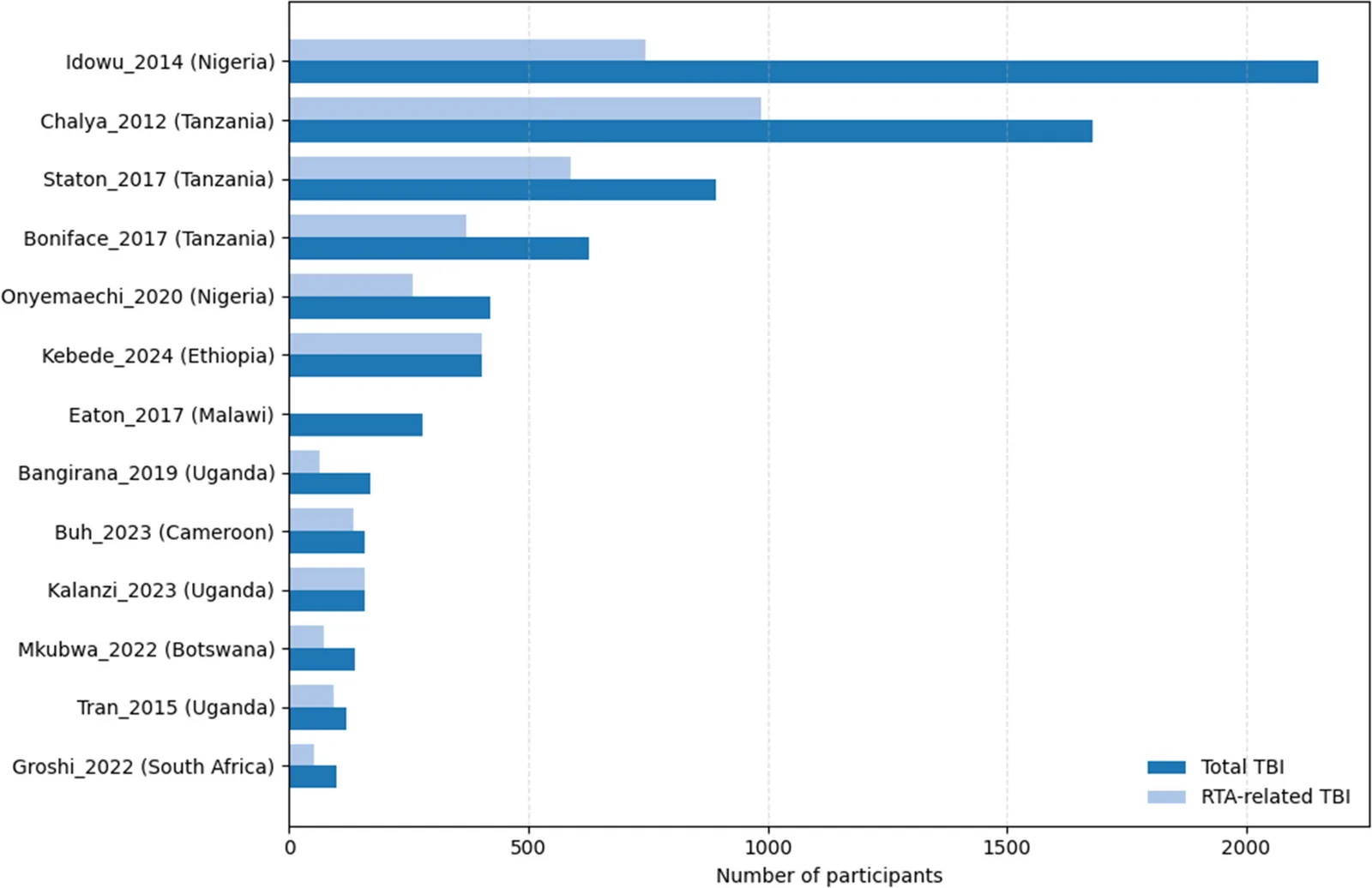
Sample sizes of included studies across African urban centers, demonstrating heterogeneity in participant numbers

Sample sizes of the included studies are illustrated in Fig. 2.

### Reported outcomes

Outcome reporting was significantly heterogeneous across studies. A few studies reported on in-hospital or ICU mortality only (Staton, Mkubwa, Kebede), others evaluated functional outcome at discharge with the Glasgow Outcome Scale (GOS) (Buh, Boniface), and one single study investigated long-term neuropsychological outcomes at six months (Bangirana). No study applied a consistent follow-up duration or outcome definition.

Severe TBI mortality ranged from 46–47% where specifically reported (Staton 2017, Buh 2023). Overall mortality across studies varied from 10.7% (Onyemaechi 2020, 1-year follow-up) to 35% (Mkubwa 2022, ICU cohort). Mortality was high, ranging from 30% to over 50% [14, 20, 26] for severe TBI (GCS ≤ 8). Glasgow Outcome Scale (GOS), which measured functional outcomes, showed a significant proportion of survivors experienced moderate to severe disability [12, 14]. In one of the studies, Neuropsychological outcomes (cognitive impairment, depression) were assessed, which found significant cognitive impairment and depression at six-month follow-up [10]. Other common outcomes included prolonged length of hospital stay and a high rate of discharge against medical advice (DAMA) [15, 17, 22].

**Fig. 3:**
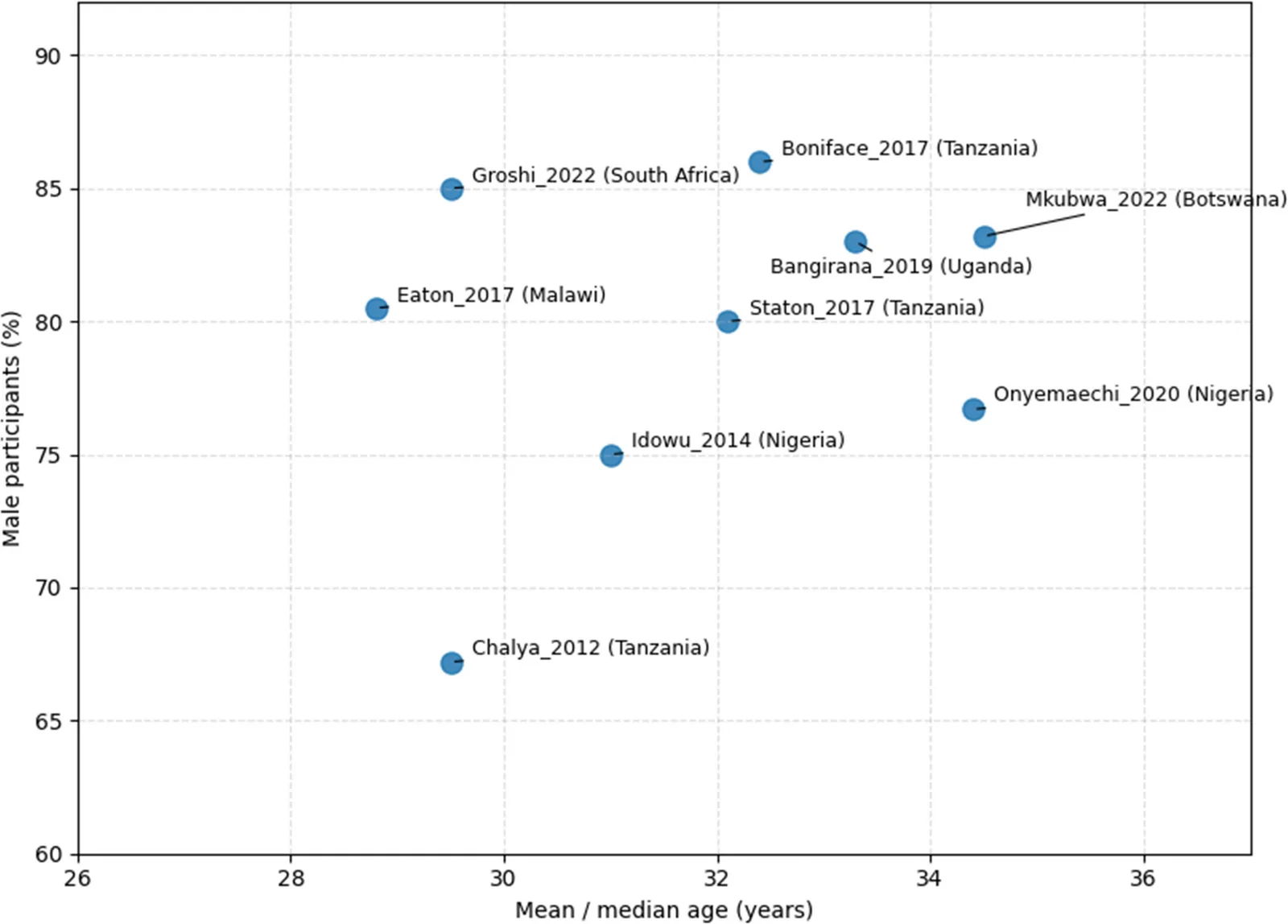
Participant demographics of included studies, showing the distribution of mean/median age and proportion of male patients

Participant demographics, including age distribution and male predominance across studies, are presented in Fig. 3.

### Predictors of outcome

A low Glasgow Coma Scale (GCS) score on admission, hypoxia, older age, abnormal pupillary reactivity, severe injury (ISS > 15), polytrauma, and the presence of hypotension were considered as key clinical predictors of poor outcome (Groshi 2022, Chalya 2012, Kebede 2024, Onyemaechi 2020). Protective factors included helmet and seatbelt use (Onyemaechi 2020, Kalanzi 2023).

Critical system-level predictors were delayed presentation (many patients arrived > 6–24 h after injury) to a definitive care facility, lack of access to computed tomography (CT) scanning, unavailability of immediate neurosurgical intervention [12, 14, 21], and DAMA due to financial constraints [15, 17, 18]. The capacity to provide neurosurgical intervention was a significant positive predictor of survival (Groshi 2022, Tran 2015).

### Patterns and differences across countries

Studies indicate that disparities in patient outcomes were significantly influenced by the distribution of healthcare system resources. Notably, mortality rates were demonstrably lower, and better functional outcomes were reported in tertiary care centers equipped with neurosurgical capabilities (Groshi 2022, Tran 2015). In contrast, facilities without such dedicated units, such as those described in studies from Tanzania and Cameroon, experienced less favorable results (Staton 2017, Boniface 2017, Buh 2023). However, these observations are associative and may reflect referral, selection, and case-mix differences rather than a causal effect of neurosurgical availability.

A further dimension of this resource disparity is the phenomenon of discharge against medical advice, which emerged as a recurrent and significant issue in studies from Ethiopia, Tanzania, and Nigeria [15, 17, 18, 22]; this decision was frequently driven by patients' financial constraints and the burden of catastrophic out-of-pocket expenses. The specific etiology of TBI also varied by location, with one large Nigerian study reporting assault as the leading cause, challenging the assumption that RTAs are universally the primary mechanism in urban settings [22].

Key clinical details, outcomes, and health system factors are summarized in Table 2.

**Table 2:** Clinical Details, Outcomes, and Health System Factors

**Author(s), Year**	**Key Clinical Characteristics**	**Severe TBI (%)**	**Primary Outcome Reported**	**Outcome Time Point**	**Health System Factors**	**Key Results**
Staton et al., 2017 [12]	66% RTAs; motorcycle 49% of RTAs; 1 intubation; limited oxygen; registry data	12.9	In-hospital mortality; GOS at discharge	Discharge	Limited secondary-injury care; inconsistent CT/intubation	Severe TBI high mortality (47%); resource gaps in secondary care prominent; females better functional recovery
Groshi & Enicker, 2022 [13]	Median post-resuscitation GCS 6; hypotension 38%; all intubated; ICP monitoring 76%	100	Functional outcome (GOS: favorable vs unfavorable)	At discharge	CT, ICU, and neurosurgical services available	Hypotension, refractory intracranial hypertension, and female sex associated with worse outcomes
Buh et al. (Fola), 2023 [14]	Median GCS 12; CT performed in 78%; intracranial lesions in 64%	24	In-hospital mortality; 6-mo GOSE	6 months	CT performed in most; financial barriers common; DAMA 33%; minimal rehab	Severe TBI mortality 46%; financial constraints and DAMA associated with worse outcomes
Boniface et al., 2017 [15]	GCS often unassessed; CT used in 85.6% of moderate/severe cases	17.8	In-hospital mortality	In-hospital	Poor prehospital care; limited trauma protocols; CT relatively high	Severe TBI and intracranial hemorrhage strongly associated with mortality
Kalanzi et al., 2023 [16]	GCS 3–8 in cohort; helmet use 19.2%; transport modes: police 40%, ambulance 36.9%	100	Injury patterns and polytrauma	Admission/in-hospital	Limited neurosurgical services; low helmet use	Severe TBI cohort; high polytrauma; vehicle crashes associated with more polytrauma than motorcycles
Chalya et al., 2012 [17]	Severe TBI 9.7%; no prehospital care; helmet 24.7%; seatbelt 13.5%	9.7	In-hospital mortality	In-hospital	Tertiary center; CT and neurosurgical capacity	Severe trauma, hypotension, and severe head injury predicted mortality
Eaton et al., 2017 [20]	Median GCS ~ 12; limited CT/ICU; neurosurgery scarce	33	Functional outcome (GOS)	Discharge	Limited CT and ICU availability; scarce neurosurgery	Severe TBI predicted poor outcome (HR 2.98)
Mkubwa et al., 2022 [25]	GCS < 8 in 45%; CT findings: SDH 38.7%, skull base fracture 27%	91	ICU mortality; functional outcome (GOS)	ICU/in-hospital	CT available; limited ICU capacity	Lower GCS, abnormal pupils, and SDH predicted mortality/time-to-death
Bangirana et al., 2019 [19]	Admission GCS strongly predictive; CT 73.6% positive; helmet 30.5%	NR	Neurocognitive impairment; depression; PTSS	6 months	Tertiary hospital; moderate CT access	Low admission GCS associated with worse neuropsych outcomes at 6 months
Kebede et al., 2024 [18]	Head injury ~ 32%; polytrauma 16%; SPO₂ < 95% in 16%	NR	In-hospital mortality	Up to 30 days	ICU admission analyzed; regional hospitals	Low GCS, hypotension, low SPO₂, complications predicted mortality
Onyemaechi et al., 2020 [21]	Mean ISS 9.2 ± 2.9; 60.1% ISS > 15; TBI 20%; delayed arrival ~ 23 h	20	Mortality; functional outcome (GOS)	1 year	Delayed presentation; limited prehospital care	1-year mortality 10.7%; TBI and high ISS predicted death; DAMA 15.9%
Idowu & Akinbo, 2014 [22]	GCS recorded; 93.2% neurotrauma; high referral burden	93.2	Functional outcome (GOS)	In-hospital/ER	Limited ICU beds; frequent inter-facility referrals	RTAs and assault were leading causes; high referral/resuscitation reflects system limits
Tran et al., 2015 [23]	All GCS ≤ 8; intracranial hematoma common	100	In-hospital mortality; functional outcome (GOS)	In-hospital	CT and neurosurgical services available	In-hospital mortality 25.8%; admission GCS predicted poor outcome

Outcome definitions, measurement tools, and follow-up time varied across studies and included in-hospital mortality, ICU mortality, functional outcomes at discharge (GOS), and longer-term neuropsychological outcomes.

**Fig. 4:**
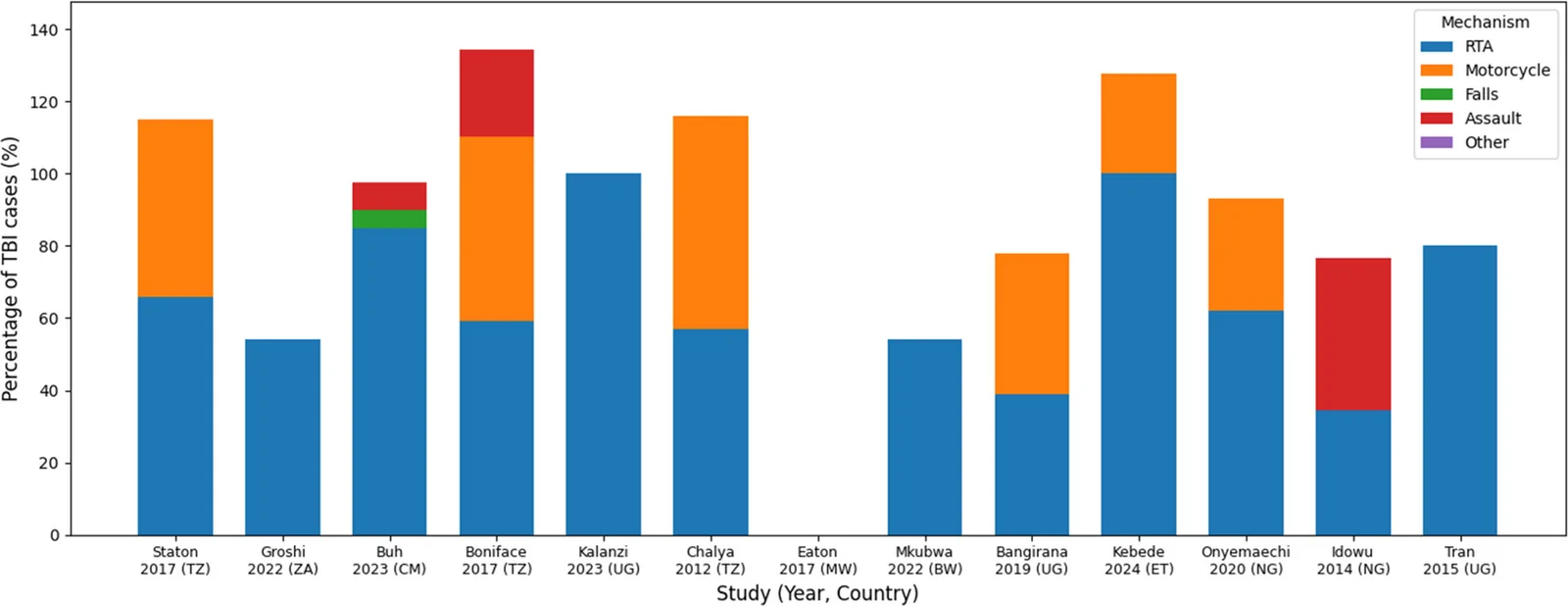
Reported causes of traumatic brain injury (TBI) across included studies, with road traffic accidents as the predominant cause, followed by motorcycle-specific, falls, interpersonal violence, and other mechanisms

The distribution of TBI causes across studies is summarized in Fig. 4.

### Risk of bias assessment

The quality of the included studies was assessed for risk of bias across all 13 studies using the Newcastle–Ottawa Scale (NOS), with an adapted version applied for the cross-sectional study. Overall, the methodological quality of the included evidence was moderate. Eleven studies were judged to have a moderate risk of bias, while two studies were rated as having a high risk of bias. None of the included studies was classified as low risk of bias. Table 1 summarizes the overall study-level risk-of-bias judgments. The use of a single unified assessment tool (NOS) ensured consistency in evaluation across study designs, including cohort, registry, and cross-sectional studies.

Most studies were generally well-conducted in terms of selection, with clear case definitions and adequate descriptions of demographic and clinical characteristics. However, several recurring methodological limitations were identified across the included studies. Outcome assessment was frequently restricted to in-hospital or discharge status, with only a small number of studies reporting longer-term follow-up (up to six months), thereby limiting interpretation of sustained functional outcomes. Adjustment for confounding was inconsistently performed; while a few studies applied multivariable analyses for key prognostic factors such as age, hypotension, injury severity, and prehospital delay, most relied on unadjusted or minimally adjusted analyses. In addition, several studies were limited by single-centre designs and modest sample sizes, which may affect both internal and external validity.

The highest risk of bias was observed in studies with more limited methodological control and weaker adjustment for confounding. For example, Tran et al. (2015) demonstrated moderate-to-high risk due to its retrospective chart review design, incomplete clinical documentation, and limited adjustment for key prognostic variables. Kalanzi et al. (2023), while methodologically constrained by its cross-sectional design, also showed limitations due to the absence of outcome follow-up and restricted analytical depth. In contrast, larger hospital-based cohort studies such as Chalya et al. (2012) and Kebede et al. (2024) provided more robust descriptive data and stronger outcome reporting, although they still lacked comprehensive adjustment for confounders and long-term follow-up.

Overall, despite variability in methodological quality, most studies were rated as having a moderate risk of bias under the Newcastle–Ottawa Scale, with a smaller proportion achieving high-quality ratings based on stronger cohort design, outcome ascertainment, and statistical adjustment.

**Fig. 5:**
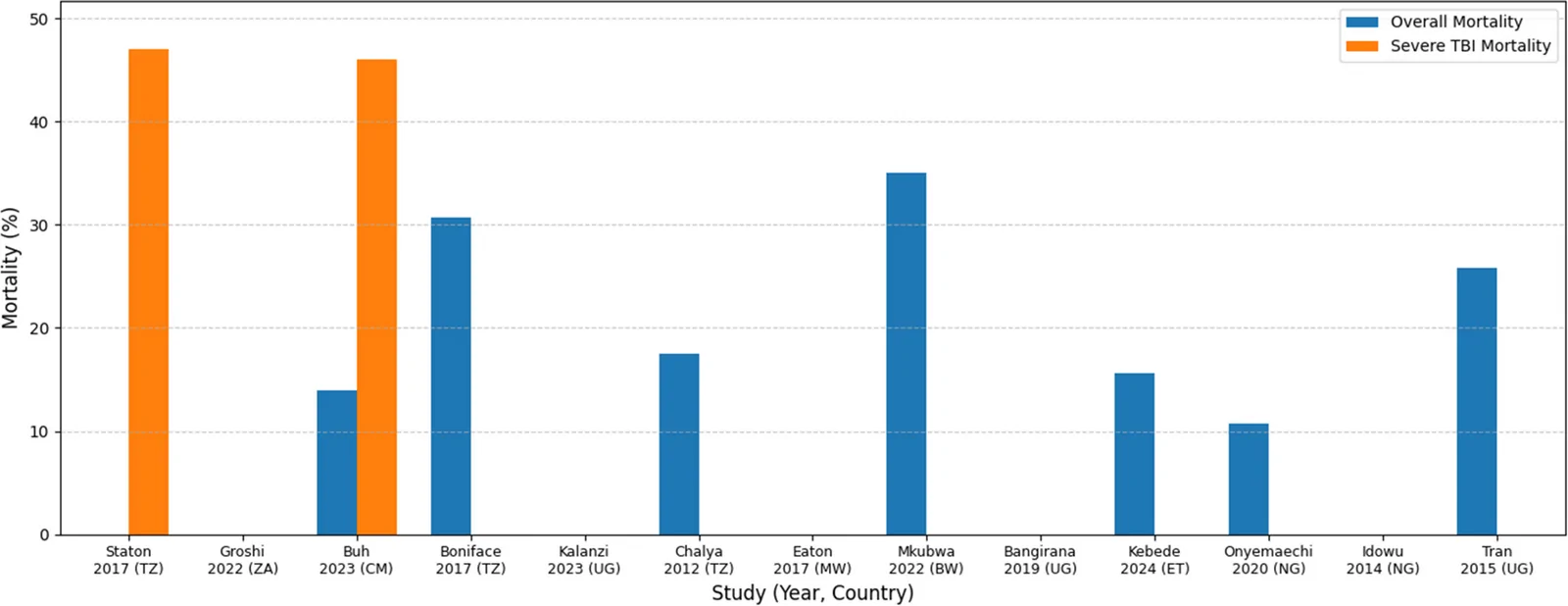
Mortality outcomes across included studies

Mortality outcomes across included studies are shown in Fig. 5.

## Discussion

### Principal findings

This systematic review establishes that Road Traffic Accidents (RTAs) remain the primary cause of Traumatic Brain Injury (TBI) in African urban centres, disproportionately affecting young males, who constitute the most active segment of the population in terms of mobility and occupational exposure. The outcomes vary considerably with injury severity. Severe TBI, defined as a Glasgow Coma Scale (GCS) score of 3–8, was associated with hospital mortality rates of 30–50%, while mild TBI generally showed more short-term recovery [27]. However, long-term follow-up data revealed that only a minority of patients achieved full recovery, while the others had significant functional and neuropsychological impairment.

These findings indicate that both injury severity and health-system constraints play a significant role in determining patients’ outcomes. While low GCS scores at presentation remain the strongest predictor of poor prognosis, other systemic limitations, including delayed access to definitive care, lack of access to computed tomography scanning, and limited neurosurgical capacity, substantially worsen outcomes. Financial constraints also resulted in high rates of Discharge Against Medical Advice (DAMA), further compromising patient recovery. Such constraints not only exacerbate early mortality but also contribute to the long-term burden of disability among survivors.

### Comparison with prior literature

The epidemiology of TBI differs substantially between high-income countries (HICs) and low- and middle-income countries (LMICs), including sub-Saharan Africa. In HICs such as the United States, Canada, and several European countries, falls, particularly among older adults, are now the leading cause of TBI, reflecting population ageing and improved road safety systems. Global estimates suggest that falls account for a large proportion of TBI cases across many countries, particularly in high-income regions [28, 29].

In contrast, in African settings such as Nigeria, Tanzania, Uganda, and Cameroon, RTAs, particularly motorcycle-related crashes, remain the predominant mechanism of injury, with young adult males most frequently affected [30]. This pattern reflects structural and contextual factors, including rapid motorisation, inconsistent enforcement of road safety regulations, low helmet use, and limited prehospital trauma systems [31].

Differences in trauma system capacity further explain outcome disparities. HICs benefit from well-developed emergency medical services, rapid transport systems, and widespread access to neurosurgical and neurocritical care, including intracranial pressure monitoring and advanced critical care interventions [32, 33]. In contrast, many African settings face delays in prehospital transport, limited imaging availability, shortages of the neurosurgical workforce, and restricted intensive care capacity. These systemic limitations contribute to the higher mortality observed in LMICs, where severe TBI mortality may be substantially higher than in HIC settings [34].

These findings reinforce the importance of context-specific epidemiological evidence. Reliance on data from HICs risks underestimating the burden and distinct injury patterns seen in rapidly urbanising African populations [35]. Existing regional literature similarly highlights health-system constraints as major determinants of outcomes and supports the need for improved surveillance and multicentre prospective data collection [36, 37].

### Health-system drivers of outcome

Variation in outcomes across studies appears to reflect differences in trauma system capacity in addition to injury severity. Studies conducted in settings with more developed tertiary services, better CT availability, and on-site neurosurgical capacity (e.g., South Africa and selected centres in Uganda and Tanzania) generally reported more favourable in-hospital outcomes compared with settings with limited imaging, delayed presentation, and constrained critical care capacity.

Financial barriers were a recurrent feature across studies and contributed to DAMA, particularly where patients were required to pay out-of-pocket for imaging, surgery, or intensive care. DAMA likely contributes to worse outcomes, including preventable mortality and long-term disability [38]. However, comparisons across studies should be interpreted cautiously due to heterogeneity in case mix, referral patterns, and outcome definitions.

The absence of standardized trauma registries and uniform outcome reporting further limits comparability. Variability in the use of GCS, GOS/GOSE, and differing follow-up time points complicates synthesis and underscores the need for harmonised reporting frameworks in African trauma research [36, 37].

### Long-term outcomes and neuropsychological burden

Traumatic brain injury resulting from road traffic accidents has been associated with persistent neurocognitive and neuropsychological sequelae, including impairments in memory, attention, learning, and executive function, as reported across several included studies. These findings highlight that the burden of TBI extends beyond acute mortality and functional status at discharge. Consequently, longer-term follow-up beyond the immediate hospitalization period, ideally extending to at least six months post-injury, is important for capturing delayed or persistent neurocognitive complications that may not be evident in early outcome assessments. In addition, psychometric and neuropsychological assessments, including tests of visual function, memory, and learning, have been suggested as useful tools for evaluating functional recovery and guiding rehabilitation planning in affected patients [39].

Across the included studies, delayed presentation to hospital care was a recurrent finding and has been attributed to prehospital and transportation-related constraints, including limited emergency medical services and infrastructural challenges. Such delays may contribute to secondary brain injury and worse clinical outcomes. Similarly, constraints in healthcare infrastructure, including limited bed capacity and restricted availability of operating theatres, were reported as barriers to timely and effective neurosurgical management in several settings, potentially exacerbating morbidity and mortality [40].

In addition, limited access to computed tomography (CT) imaging was frequently reported across studies and was attributed to shortages of functioning equipment, equipment downtime, and inadequate maintenance systems. These constraints may delay diagnosis and definitive management of intracranial injuries. Strengthening imaging capacity through improved maintenance systems and expanded access in both public and private healthcare facilities has been suggested as a potential strategy to address this gap [41–43].

### Strengths and limitations of the study

The included studies demonstrated several strengths, including multi-country representation across sub-Saharan Africa and consistent identification of key clinical prognostic indicators such as low Glasgow Coma Scale (GCS) score, hypotension, and hypoxia. These recurring findings enhance the internal consistency of the evidence base despite methodological heterogeneity.

However, important limitations were identified. Many studies were single-centre and hospital-based, which may introduce selection bias toward more severely injured patients and limit generalisability. The majority of studies were retrospective in design, with inherent risks of missing data and measurement bias. Outcome assessment was frequently restricted to in-hospital or short-term endpoints, and variation in outcome definitions limited comparability across studies.

In addition, several studies included mixed-mechanism traumatic brain injury cohorts without stratified reporting for road traffic accident–related cases. This may have introduced exposure misclassification and potentially diluted mechanism-specific effect estimates. Consequently, the findings should be interpreted as reflecting urban African traumatic brain injury populations in which road traffic accidents are the predominant injury mechanism, rather than being exclusively specific to RTA-related traumatic brain injury.

Finally, differences in health-system capacity, case mix, and referral pathways across studies limit direct comparability and complicate causal interpretation of observed associations between system-level factors and outcomes.

### Clinical and research implications

The findings emphasise the importance of early physiological stabilisation, rapid neuroimaging, and timely neurosurgical intervention in improving outcomes following TBI. However, observed associations between health-system capacity and outcomes should be interpreted cautiously, as most studies were observational and subject to confounding and selection bias.

Future research should prioritise prospective multicentre cohort studies with standardized reporting of injury severity, management pathways, and functional outcomes. Longer-term follow-up (≥ 6 months) using standardized tools such as GOSE is recommended where feasible, although pragmatic short-term outcome measures may also be necessary in resource-limited contexts. Additional research is needed to improve the understanding of economic barriers, care pathways, and scalable system-level interventions.

## Conclusion

Traumatic brain injury remains a major cause of mortality and disability in African urban centres, with road traffic accidents representing a leading mechanism of injury in most included cohorts. Poor outcomes were consistently associated with severe injury, delayed presentation, limited access to imaging and neurocritical care, and financial barriers to treatment. However, the evidence base is constrained by predominantly observational single-centre studies, heterogeneous outcome reporting, and limited long-term follow-up. Future multicentre prospective studies using standardized outcome measures are needed to better characterize the burden of TBI and evaluate interventions aimed at improving trauma care and patient outcomes across African settings.

## Supplementary Information

Supplementary Material 1

## Data Availability

Data sharing is not applicable to this article as no datasets were generated or analysed during the current study. All data extracted and analysed in this systematic review are derived from publicly available published studies cited in the manuscript.

## Abbreviations

CT: Computed Tomography
DAMA: Discharge Against Medical Advice
GCS: Glasgow Coma Scale
GOS: Glasgow Outcome Scale
ICU: Intensive Care Unit
LMIC: Low- and Middle-Income Countries
MeSH: Medical Subject Headings
PRISMA: Preferred Reporting Items for Systematic Reviews and Meta-Analyses
PROSPERO: International Prospective Register of Systematic Reviews
RoB: Risk of Bias
RTA: Road Traffic Accident
TBI: Traumatic Brain Injury
