# Supplementary material for: Traumatic brain injury in African urban centers: The role of road traffic accidents, outcomes, and health-system factors: a systematic review
Source: BMC Neurol. 2026 Aug 8;26:701. doi: 10.1186/s12883-026-05263-z (PMC13733039; doi:10.1186/s12883-026-05263-z)
Supplement: Supplementary file 1 [file 12883_2026_5263_MOESM1_ESM.docx]

**Supplementary File 1**

# **Search Strategy**

A comprehensive systematic literature search was conducted in PubMed, Google Scholar, and relevant African trauma registries from 1 January 2010 to 18 May 2025. The search strategy combined Medical Subject Headings (MeSH) and free-text keywords, including synonyms and controlled vocabulary tailored to each database. The strategy was developed following the PRESS (Peer Review of Electronic Search Strategies) guideline to ensure completeness and reproducibility.

Key search concepts included:

- “Traumatic Brain Injury” OR “TBI”
- “Road Traffic Accidents” OR “Motor Vehicle Accidents”
- “Neurosurgery” OR “Neurosurgical Trauma”
- “Africa” OR individual African country names
- “Urban centers” OR “city” OR “metropolitan”
- “Outcomes” OR “mortality” OR “Glasgow Outcome Score”

Boolean operators (AND/OR) were used to combine search concepts appropriately.

# **Study Selection Process**

All retrieved records were imported into a reference manager, and duplicates were removed. Titles and abstracts were screened for relevance, followed by full-text review based on eligibility criteria. The first 300 records identified in Google Scholar were screened due to their large yield and limited advanced filtering capabilities. Screening was conducted independently by two reviewers, with disagreements resolved through discussion.

# **Appendix A: Full Search Strategy**

## **A1. PubMed Search String**

("Traumatic Brain Injury"[MeSH Terms] OR "traumatic brain injury" OR TBI OR "head injury")

AND

("Motor Vehicle Accidents"[MeSH Terms] OR "road traffic accident*" OR "road traffic crash*" OR "motor vehicle accident*" OR RTC OR MVC)

AND

("Neurosurgery" OR "neurosurgical trauma" OR "head trauma management")

AND

("Africa"[MeSH Terms] OR Africa OR Nigeria OR Uganda OR Tanzania OR Kenya OR Ethiopia OR Cameroon OR Malawi OR Botswana OR "sub-Saharan Africa")

AND

("urban" OR "urban center*" OR city OR cities OR metropolitan)

AND

(outcome* OR mortality OR "Glasgow Outcome Scale" OR GOS OR disability)

## **A2. Google Scholar Search Strategy**

Due to limited Boolean functionality in Google Scholar, simplified keyword combinations were used:

"traumatic brain injury" AND "road traffic accident" AND Africa AND (outcome OR mortality OR "Glasgow outcome scale")

Additional targeted searches were performed using combinations of:

- “TBI Africa mortality”
- “road traffic injury neurosurgery Africa outcomes”
- “traumatic brain injury urban hospitals Africa”

The first 300 results were screened for relevance, and duplicates or irrelevant citations were excluded.

## **A3. African Trauma Registries**

Where available, institutional and regional trauma registry databases were searched using the same keyword combinations:

- Trauma registry reports
- Neurosurgical audit datasets
- Hospital-based injury surveillance systems in Sub-Saharan Africa

# **Notes on Replicability**

- Search strategy was adapted per database syntax
- English language restrictions were applied
- Reference lists of included studies were manually screened for additional eligible studies
